# Supplementary figures and images for: Real-time resolution of short-read assembly graph using ONT long reads
Source: PLoS Comput Biol. 2021 Jan 20;17(1):e1008586. doi: 10.1371/journal.pcbi.1008586 (PMC7850483; doi:10.1371/journal.pcbi.1008586)

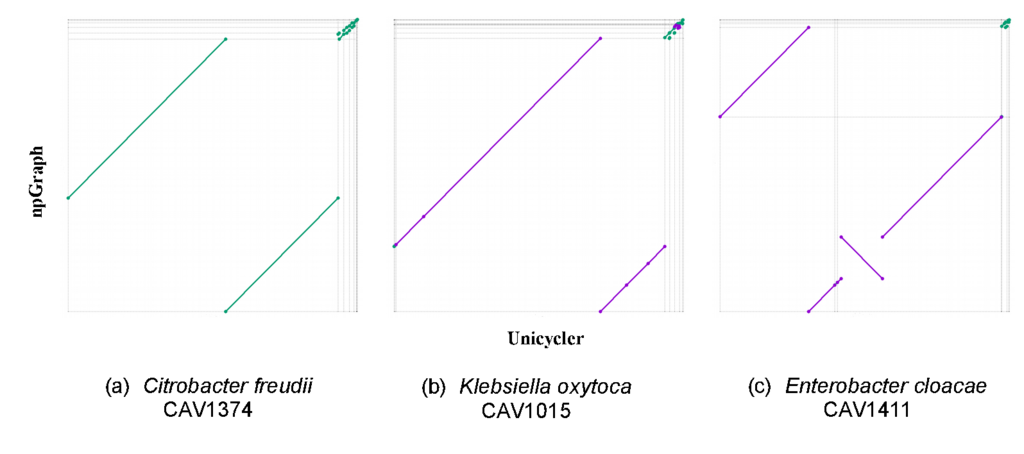

Supplement: S1 Fig — Structural agreements between two methods were found in (a) C.freundii and (b) K.oxytoca assembly contigs. On the other hand, for (c) E.cloacae sample, there was a disagreement detected between 2 largest contigs given by two assembly algorithms. (TIF) [file pcbi.1008586.s001.tif]

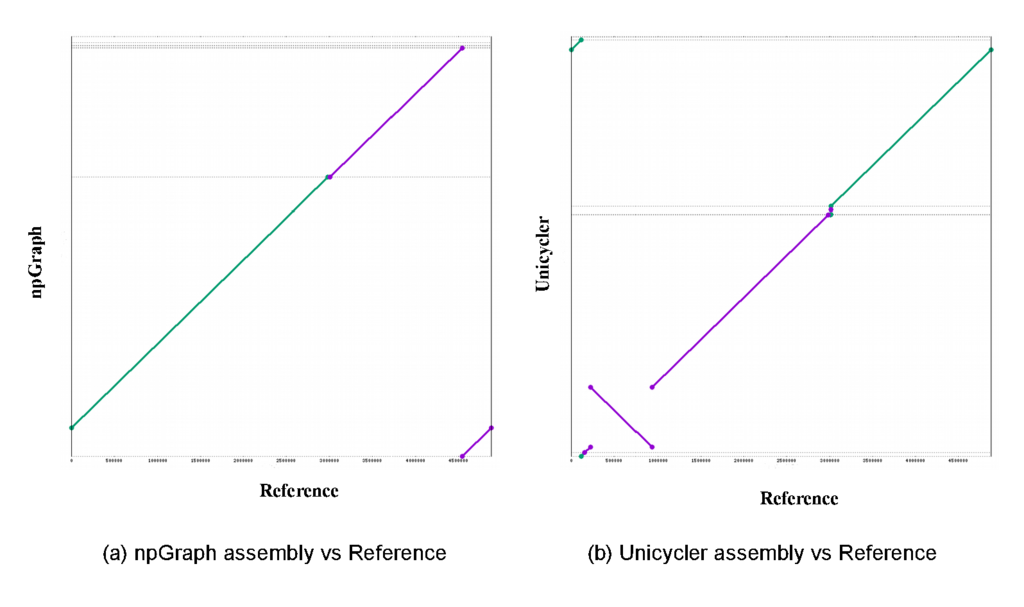

Supplement: S2 Fig — The former suggests a structural variant, the latter is virtually an 1-to-1 mapping. (TIF) [file pcbi.1008586.s002.tif]
