# Supplementary material for: Real-time resolution of short-read assembly graph using ONT long reads
Source: PLoS Comput Biol. 2021 Jan 20;17(1):e1008586. doi: 10.1371/journal.pcbi.1008586 (PMC7850483; doi:10.1371/journal.pcbi.1008586)
Supplement: Attachment — Submitted filename: Response_Letter.docx [file pcbi.1008586.s005.docx]

# Response to Reviewer #1:

Comment:
Reviewer #1: The manuscript describes the algorithm, npGraph, offers a way to improve the assembly completeness and visualize the procedure in real-time using Nanopore long reads. On the synthetic and real dataset, the authors show that npGraph yields assemblies of comparative performances to other hybrid scaffolders. It is really a nice tool, which is thought to be popular in sequencing analysis. To make the results stronger in comparison, I give my suggestions and questions.

Response:

We thank the reviewer for their comment.

Comment:

1) *In Contigs Binning step, by using kmer coverages to determine the contigs membership, is the kmer size sensitive to this?*

Response:

We only use the SPAdes optimized value of *kmer* (normally k=127) for our pipeline and the estimation only apply for contigs with significant length.

In addition, topology information from the assembly graph has been used afterward to reduce the number of mis-classified cases.

Comment:

2) *npGraph is a hybrid scaffolder based on streaming data. In table 1, Unicycler has high accurate assemblies than other algorithms, even it takes long time. Though with unique merits by itself, it would be good to show the performances and benchmarks among hybrid scaffolders with increasing coverage of long reads (Such as 1x -> 5x -> 10x -> 20x).*

Response:

While this analysis is of interest, in this manuscript we have set out to focus on truly streaming analysis, which Unicycler does not support. We feel that the paper shows conclusively that npGraph produces assemblies which are as good as Unicycler, while also providing the ability to carry out analysis in a streaming manner.

Comment:

3) *Another two potentially missing software used in scaffolding would be FastSG (https://github.com/adigenova/fast-sg) and* *LRScaf (https://github.com/shingocat/lrscaf), which are two differences strategies on hybrid scaffolding. FastSG does the scaffolding step by simulating mate-pair information from long reads, whereas LRScaf is fast hybrid scaffolders on low coverage long reads by using full length information.*

Response:

Follow the reviewer’s suggestion we attempted to benchmark against both FastSG and LRscaf. Unfortunately, despite our best attempts we were unable to install and run FastSG workflow on our HPC system.

However, LRScaf is more straight-forward to use and is also fast to run. We included the results of LRScaf with default parameters using SPAdes assembly and minimap2 alignments in the Supplementary Table S1.

We added the following text in the results section:

“LRscaf has been designed as a computationally efficient hybrid assembly tool which is scalable to large genomes. We ran LRScaf using default parameters on the extended benchmarking set presented in Supplementary table 1. We observed that LRScaf assemblies had fewer misassemblies than npScarf, and also exhibited a low mismatch and indel rate, but produced more fragmented assemblies than either npGraph or unicycler.”

Comment:

4) *As mentioned by authors, npScarf is a greedy scaffolder with only 1 spanning long-read, whereas npGraph requires 3 reads. If improving the required number of long reads for npScarf, would the misassemblies and contiguous of assemblies be decreased? It is reasonable to benchmark the performance between npScarf and npGraph on similar circumstances.*

Response:

Even though require only 1 supported read to form the initial bridge, npScarf implemented a self-correction mechanism in which an existing bridge could be parted and reformed using different pair of anchors. Increasing this parameter can affect immediate results but would not much in long term. npGraph, on the other hand, implements an irreversible approach thus requires more supporting reads to start with. The difference setting regarding this parameter reflects 2 different strategies thus as a result, would not be equal.

Comment:

5) *Line 79, “In order to define a customised metric which is sample and fast to calculate”, is this a typo for simple?*

Response:

Typo corrected.

Comment:

6) *In supporting figure S2, the figure is not consistent with the description.*

Response:

Typos corrected.

# Response to Reviewer #2:

Comment:

*This paper reports a new streaming pipeline npGraph for hybrid assembly, which uses error-prone long reads to improve fragmented assemblies from short reads. The idea is to use long reads to bridge contigs in the assembly graph from short reads. Tested on simulated and real datasets, the proposed streaming pipeline achieved comparative performances with existing batch-mode hybrid assemblers including Unicycler and SPAdes hybrid, and outperformed the existing streaming*

*approach npScarf. The method is sound and results are convincing.*
*My only doubt about the proposed approach is the significance of having a streaming algorithm for hybrid assembler. There are important real-time analysis of the ONT reads, but I am not so sure about hybrid assembly.*

Response:

Having a streaming ONT hybrid assembly facilitates optimal use of nanopore sequencing resources – i.e prevention of over-sequencing or under-sequencing using the ONT platforms when a complete assembly is of interest. It can facilitate further gains in efficiency of hybrid assembly in combination with ReadUntil.

We have added the following text in the introduction to emphasise these applications:

“Hybrid assembly with ONT reads provides the opportunity to use long reads to fully resolve existing microbial genome assemblies. However, in order achieve this in an effective manner it is necessary to avoid both under-sequencing (and thus not completely resolving the assembly) or over-sequencing (and thus incurring unnecessary costs and time-to-results). In addition, the release of Read Until API provides the opportunity to selectively enrich parts of the genome, as has been implemented in customised targeted sequencing applications ~\cite{Payne2020}. The combination of ReadUntil with streaming hybrid assembly opens the possibility for further efficiency gains by targeting sequencing to unresolved regions of the genome.”

Comment:

1) *How the important parameters (the eps and min samples ) for DBSCAN are determined? What's their impact on the performance of npGraph.*

Response:

The default parameter for DBSCAN is set empirically on a set of significant contigs to identify the main replicon groups (equivalent to different chromosome, plasmids, taxa). It worked well with isolates or simple mixture of microbial genomes. In more complicated cases, additional curation might need to be done and/or using external binning algorithms.

Comment:

2) *I assume that the binning results are used to constraint the bridge candidates -- bridges are only considered between contigs in the same bin. This needs to be clarified in the methods.*

Response:

Bridges can be considered between contigs from different bins given that their Poisson distributions are not too far away from each other (based on the D(P1,P2) metric) and there are enough long reads supporting such connections. Binning results thus can be calibrated accordingly over time as well.

To clarify this, we add the following into Contigs binning section:

“This information can aid the bridging phase later, as member contigs of a bridge are expected to appear in the same group. However, the criteria can be relaxed between closed groups given strong connecting evidence from long read alignments.”

Comment:

3) *The paper mentioned that external binning algorithms including MetaBAT and maxBin can be utilized. Are they already implemented in the pipeline? If not, will that be straightforward to implement?*

Response:

Yes, use of external binning algorithms are supported by npGraph. However for the current study research, only isolate data sets were subjected to study and the built-in DBSCAN algorithm was good enough.

Comment:

4) *Is it a typo in algorithm 2: set of candidate paths connecting v0 to v2 -> v0 to vn?*

Response:

Typo corrected.

Comment:

5) *The relationship between Algorithm 1 & 2 is not well explained.*

Response:

We edited the section “Path finding algorithm” and re-ordered the 2 algorithms for better explanation and connection between them. Basically algorithm 1 is a sub-routine invoked inside Algorithm 2 as being pointed out in our new text:

“...Algorithm 1 will call Algorithm 2 when the base condition is met, meaning the target bridge is unbreakable (no more in-between step available)...”

Comment:

6) *It is unclear how the estimated multiplicity is used for path finding (aren't paths candidates ranked according to the likelihood computed based on long reads to contig alignment?).*

Response:

The multiplicity provides an estimation of the number of times a contig should appear in the final assembly thus it is important to assign uniqueness/repetitiveness of a contig. Also, we pointed out the role of multiplicity estimation for real-time assembly in section “Building bridges in real-time”:

“The real-time bridging method considers the dynamic aspect of multiplicity measures for each node, meaning that a n-times repetitive node might become a unique node at certain time point when its (n-1) occurrences have been already identified in other distinct unique paths.”

On the other hand, the multiplicity of shorter, repetitive contigs are less confident and only used to augment the likelihood score of a path in addition to the alignment score.

Comment:

7) *Line 79, sample and fast -> simple and fast?*

Response:

*Typo corrected.*
