# Supplementary material for: Real-time resolution of short-read assembly graph using ONT long reads
Source: PLoS Comput Biol. 2021 Jan 20;17(1):e1008586. doi: 10.1371/journal.pcbi.1008586 (PMC7850483; doi:10.1371/journal.pcbi.1008586)
Supplement: Attachment — Submitted filename: Response_Letter.docx [file pcbi.1008586.s006.docx]

# Response to Reviewer #1:

Comment: I am overall satisfied with the revised manuscript and all the concerns have been answered and resolved. npGraph gives a flexible and reliable opportunity for hybrid scaffolding with streaming data.

Response:

We thank the reviewer for their comment.

Comment:

1) However, on the table 1 benchmarks, the metrics are measured on batch-mode, I recommended to have a description of how many folds of data to yield these final assemblies.

Response:

We added the following from line 246:

“The nanopore read depth for each data set is approximately 65-fold coverage even though much less data were needed to generate these final results by the aforementioned hybrid methods.

”

Comment:

2) On line 262, there should be a blank between Unicycler and required.

Response:

Typo corrected.

Comment:

3) On line 305, the unit ‘Kbp’ is no need to italic.

```
Response:
 Typo corrected.
```

# Response to Reviewer #2:

Comment:

*The authors have addressed all my concerns.*

Response:

*We thank the reviewer for their comment.*
